# Sonographic Features of Pure Mucinous Breast Carcinoma With Micropapillary Pattern

**DOI:** 10.3389/fonc.2021.644180

**Published:** 2021-10-22

**Authors:** Wu Zhou, Yong-Zhong Li, Li-Min Gao, Di-Ming Cai

**Affiliations:** ^1^ Department of Ultrasound, West China Hospital, Sichuan University, Chengdu, China; ^2^ Department of Pathology, West China Hospital, Sichuan University, Chengdu, China

**Keywords:** breast, pure mucinous carcinoma, micropapillary, ultrasonography, mixed mucinous carcinoma

## Abstract

**Objective:**

Previous studies have mostly discussed the clinical manifestations and prognosis of mucinous breast carcinoma with a micropapillary pattern. The purposes of this study were to investigate the sonographic features of pure mucinous breast carcinoma with micropapillary pattern (MUMPC) and to identify the role of ultrasound in the differential diagnosis between MUMPC and conventional pure mucinous breast carcinoma (cPMBC).

**Materials and Methods:**

We obtained written informed consent from all patients, and the Ethics Committee of West China Hospital approved this retrospective study. The study was conducted between May and August 2020. We enrolled 133 patients with 133 breast lesions confirmed as mucinous breast carcinoma (MBC) histopathologically between January 2014 and January 2020.We retrospectively assessed sonographic features (margin, shape, internal echogenicity, calcification, posterior acoustic feature, invasive growth, blood flow grade, and rate of missed diagnosis) and clinical characteristics (age, tumor size, tumor texture, initial symptom, and lymph node metastasis). Bivariable analyses were performed using SPSS version 19.0.

**Results:**

The 133 lesions included 11 MUMPCs, 65 cPMBCs, and 57 mixed MBCs (MMBCs). There were significant differences in margin, shape, calcification, posterior acoustic feature, invasive growth, rate of missed diagnosis, average tumor size, and lymph node metastasis among the three groups (*p* < 0.05). The subsequent pairwise comparisons showed that there were significant differences in lymph node metastasis, margin, and invasive growth between MUMPC and cPMBC (*p* < 0.05). In patients aged >45 years, there was a significant difference in tumor size among the three groups (*p* = 0.045), and paired comparison showed that the average tumor size in the cPMBC group was larger than that in the MMBC group (*p* = 0.014).

**Conclusion:**

MUMPC showed a non-circumscribed margin and invasive growth more frequently than cPMBC did. Lymphatic metastasis was more likely to occur in MUMPC than cPMBC. Ultrasound is helpful to distinguish MUMPC from cPMBC.

## Introduction

Mucinous breast carcinoma (MBC) is a relatively rare entity of breast neoplasm with a characteristic of abundant extracellular mucin, representing about 1%–4% of all the primary mammary carcinomas and associated with a favorable prognosis (1, 2). In elderly patients, a slightly higher incidence rate of 6%–7% has been reported (3, 4). Pathologically, MBC is classified into two subtypes according to the degree of cellularity: pure MBC (PMBC) and mixed MBC (MMBC). PMBC consists exclusively of tumor cells responsible for mucoid production, and the mucoid component accounts for >90% of the tumor. In MMBC, 50%–90% is mainly mucinous and also admixed with an infiltrating ductal epithelial component (5). Many investigations have shown that PMBC is an indolent tumor linked with a favorable prognosis, whereas MMBC exhibits a contrasting biological behavior (6, 7). In recent years, some researchers have revealed that a small proportion of PMBCs behaved as aggressively as MMBCs (8, 9). Ranade et al. found that a micropapillary pattern was seen in 60% of lymph-node-positive PMBCs and 14% of lymph-node-negative PMBCs, which indicated that the micropapillary architecture played an important role in the development of lymph node disease (10). It appears to be particularly important to understand mucinous breast carcinomas with micropapillary pattern (MUMPCs) more profoundly. In this study, we retrospectively investigated the sonographic features of MUMPC and identified the role of ultrasound in the differential diagnosis between MUMPC and cPMBC.

## Materials and Methods

### Patients and Lesions

The Ethics Committee of West China Hospital approved this retrospective study, and written informed patient consent was obtained. The study was conducted between May and August 2020. We enrolled 135 consecutive patients who underwent surgery and were diagnosed histopathologically with MBC between January 2014 and January 2020. Two cases were excluded because one of them was male, and another had two concurrent different types of left breast carcinoma. All patients had undergone ultrasound examinations, and their medical records were available.

### Ultrasonic Imaging and Image Interpretation

Ultrasound examinations were performed using a Philips IU22 scanner (Philips Medical Solutions; Mountain View, CA, USA)with a 5–12-MHz linear transducer and Logiq E9 (GE Healthcare, Milwaukee, WI, USA) with a 5–15-MHz linear transducer. The ultrasonic equipment was operated and adjusted properly. We recorded the tumor size, location, and sonographic features (margin, shape, internal echogenicity, calcification, posterior acoustic feature, invasive growth, and blood flow grade). The Adler semiquantitative analysis of blood flow grading was performed to evaluate the intratumoral blood supply. All ultrasound data were acquired from the Picture Archiving and Communication System of the Department of Ultrasound.

All US images in the 133 patients were prospectively and independently interpreted by two radiologists (W.Y.D. and T.Z., with 8 and 6 years of experience in breast US, respectively). They had not performed the US examinations and were blinded to clinical data and pathological findings. Initially, each reader independently assessed the ultrasound parameters on each image. Subsequently, the two readers jointly reviewed the images of which they originally had different ideas and then reached an agreement on the characterization of breast US findings in those cases.

### Clinical Findings

We recorded patient age, tumor size, tumor texture, initial symptoms, lymph node status, and pathological pattern. The pathological pattern was based on the WHO Classification Standards for breast cancer, 2012. All clinical data of patients included were obtained from the Hospital Information System of West China Hospital of Sichuan University.

### Statistical Analysis

Statistical analysis was carried out using SPSS version 19.0 (IBM, Armonk, NY, USA) by a statistician with a Ph.D. from Sichuan University. The continuous data included the age of patients and tumor size. We verified whether the data were normally distributed by the Shapiro–Wilk test. Data of normal distribution were represented by mean ± standard deviation. Data that were not normally distributed were represented by the median and interquartile range (IQR). The statistical techniques used for analysis were one-way analysis of variance (ANOVA), chi-squared test or Fisher’s exact test, Kruskal–Wallis test, and Kappa test. Pairwise comparisons of the statistically significant differences among the three groups were conducted using the Student–Newman–Keuls *q* test for continuous variable or Bonferroni correction for categorical variable. The *κ* statistic was used to determine the interobserver agreement for various US parameters. We regarded the interobserver agreement as slight when *κ* was less than 0.21, fair when *κ* ranged from 0.21 to 0.40, moderate when *κ* ranged from 0.41 to 0.60, substantial when *κ* varied from 0.61 to 0.80, and almost perfect when *κ* was greater than 0.81. A two-tailed *p*-value <0.05 was considered statistically significant.

## Results

**Figure 1:**
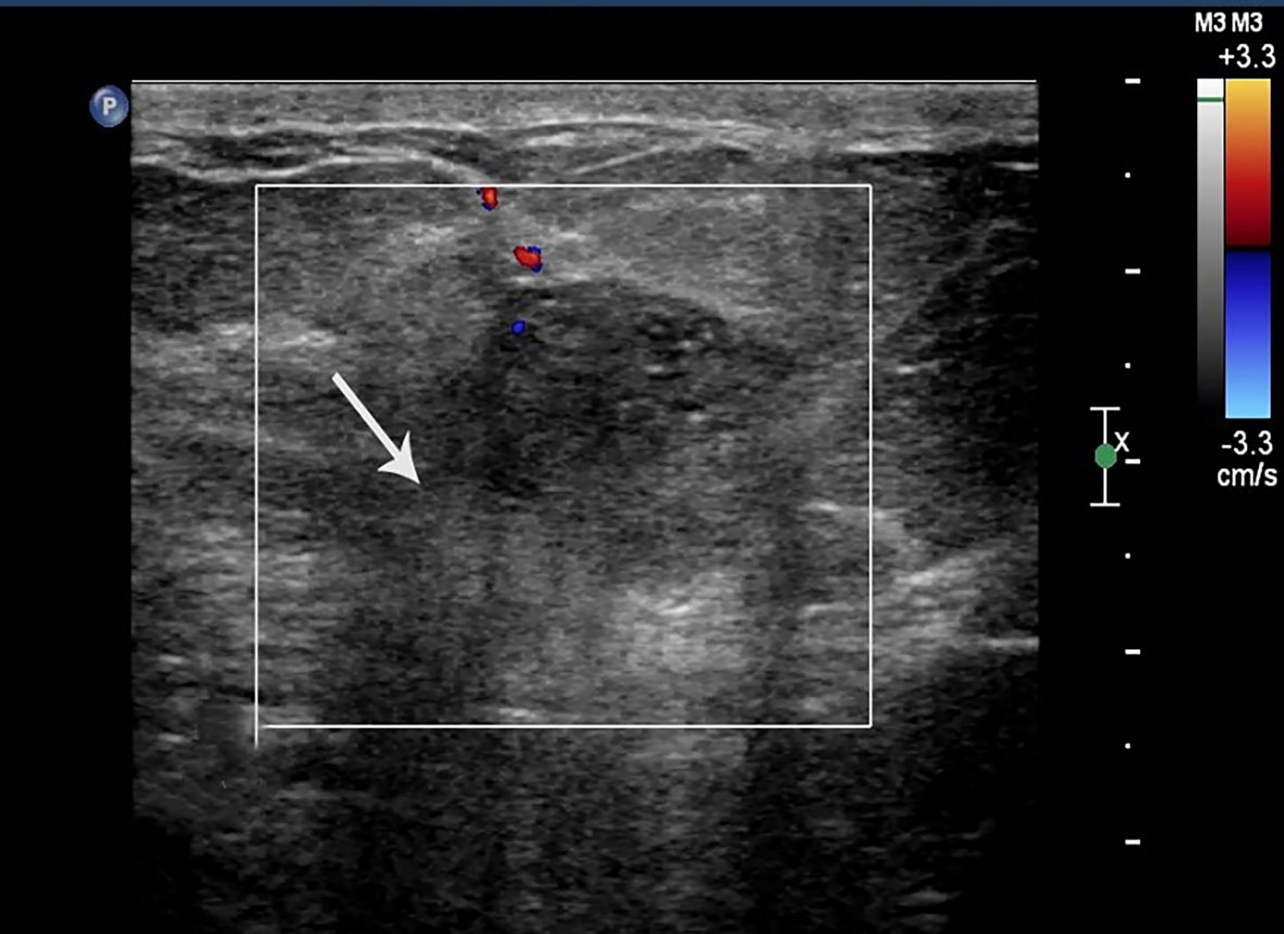
Case 2: A 55-year-old woman with MMBC presented with a hypoechoic mass (1.9 × 1.6 × 1.5cm) in the right breast. The mass presented with a non-circumscribed margin (white arrow).

**Figure 2:**
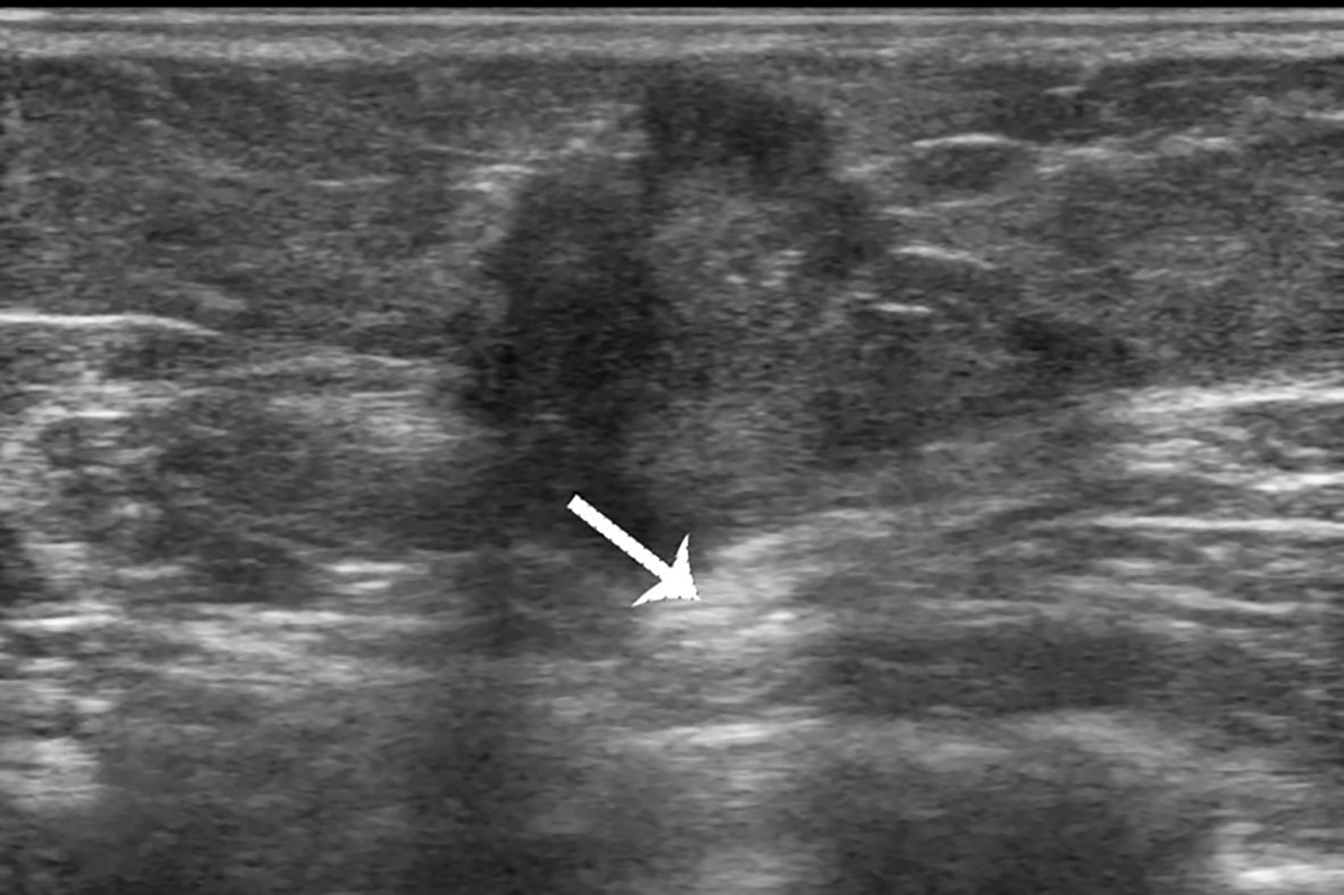
Case 1: A 37-year-old woman with mucinous breast carcinoma with micropapillary pattern (MUMPC) presented with a hypoechoic mass (1.7 × 1.5 × 1.4 cm) in the left breast. The lesion had a non-circumscribed margin, irregular shape, and enhanced posterior echo (white arrow).

**Figure 3:**
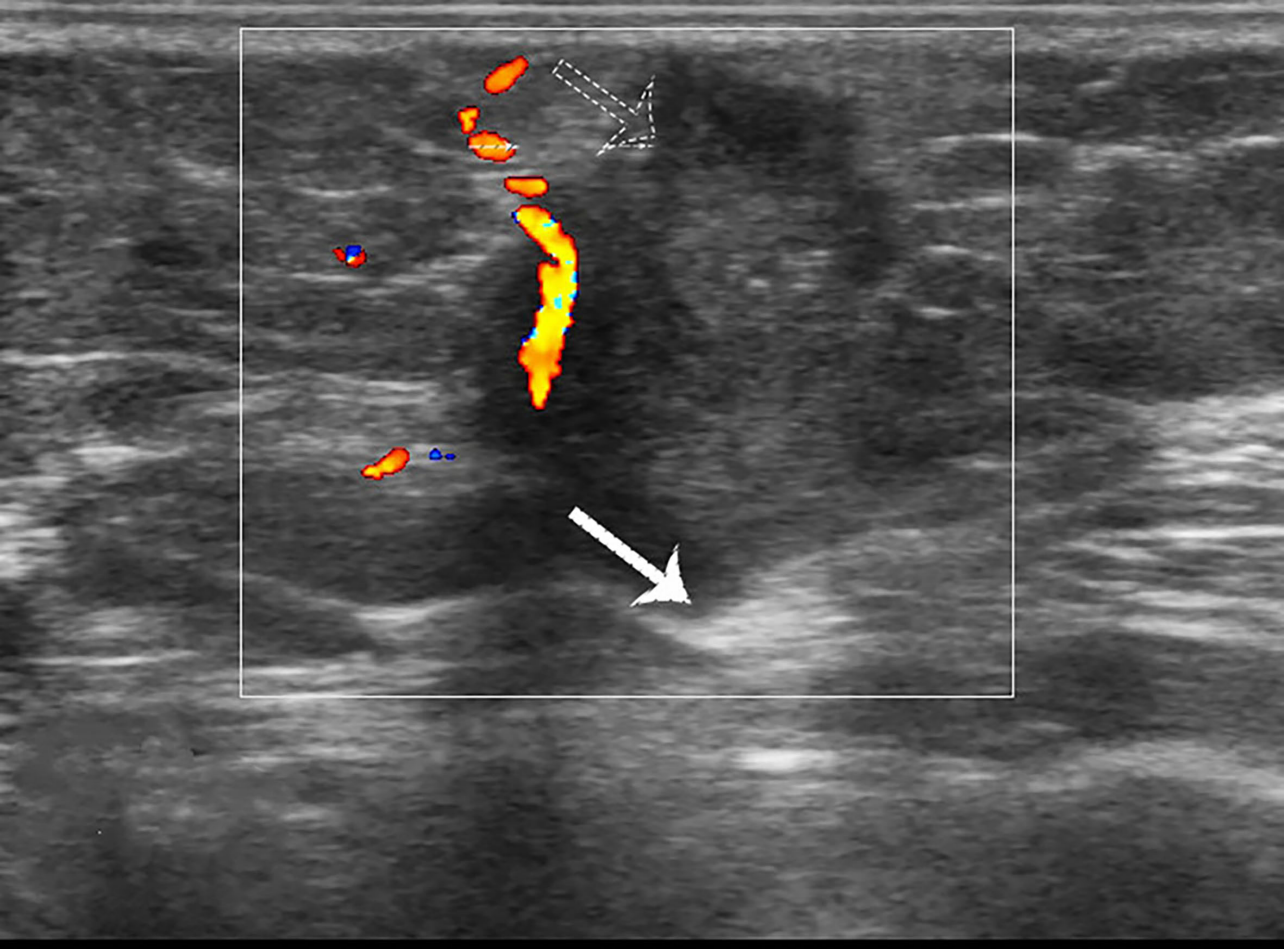
Case 1: A 37-year-old woman with MUMPC presented with a hypoechoic mass (1.7 × 1.5 × 1.4 cm) in the left breast. Subcutaneous and retromammary fat layers were both infiltrated (white and dotted arrow).

**Figure 4:**
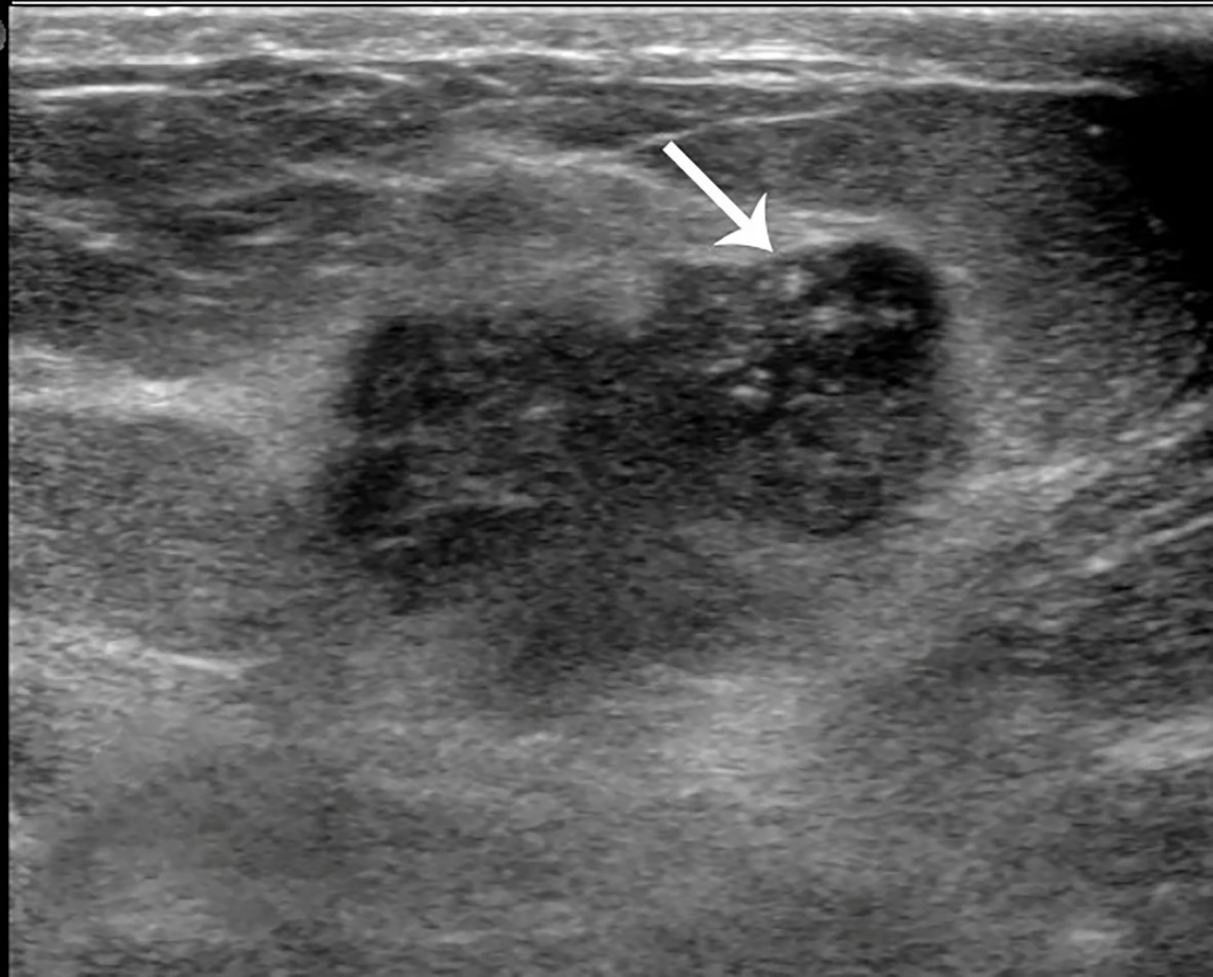
Case 2: A 55-year-old woman with mixed mucinous breast carcinoma (MMBC) presented with a hypoechoic mass (1.9 × 1.6 × 1.5cm) in the right breast. The mass presented with an irregular shape and some punctate calcifications (white arrow).

**Figure 5:**
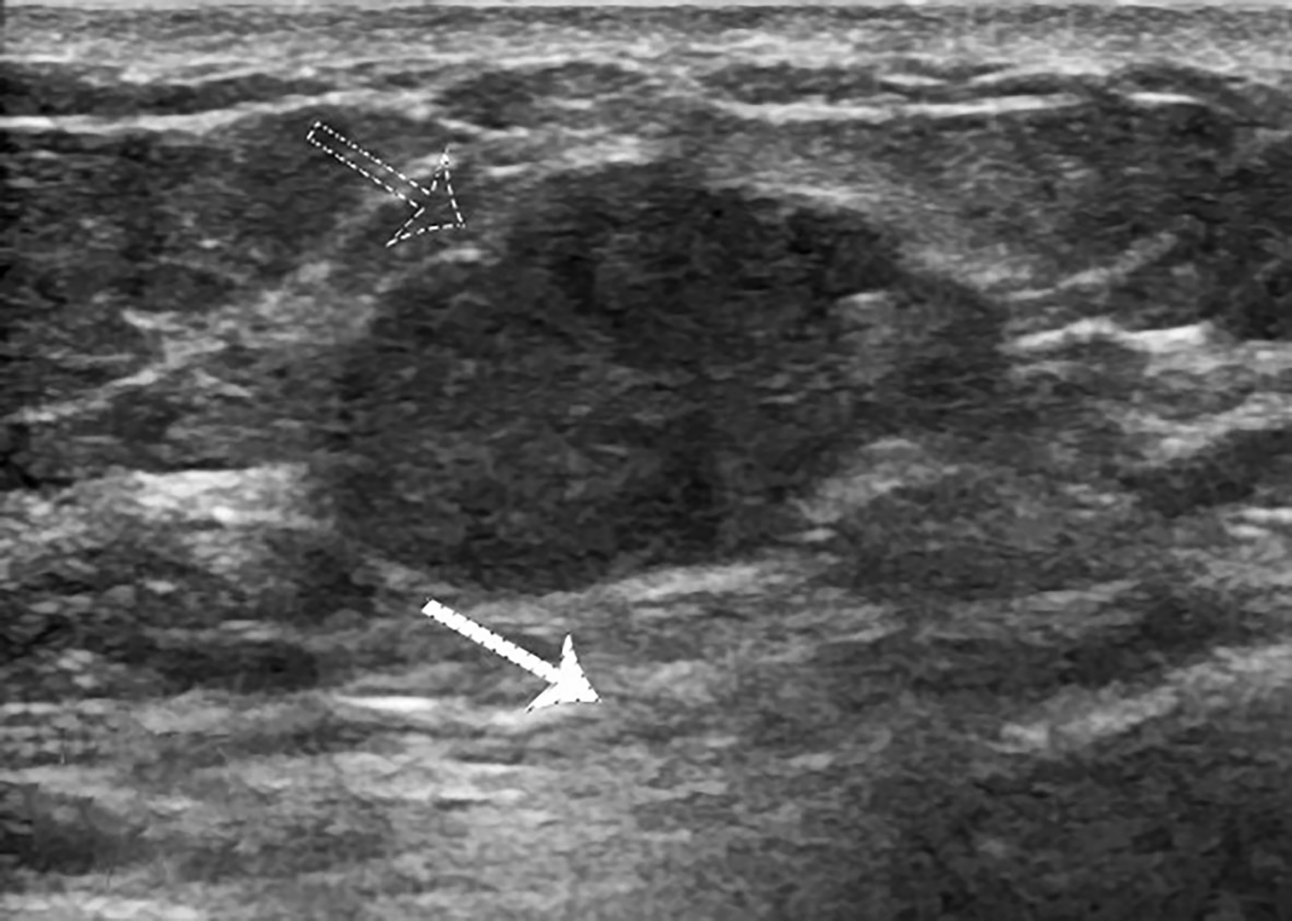
Case 3: A 49-year-old woman with conventional pure mucinous breast carcinoma (cPMBC) presented with a hypoechoic mass (1.7 × 1.4 × 1.3 cm) in the right breast. The lesion presented with a circumscribed margin (dotted arrow), irregular shape, and enhanced posterior echo (white arrow).

**Figure 6:**
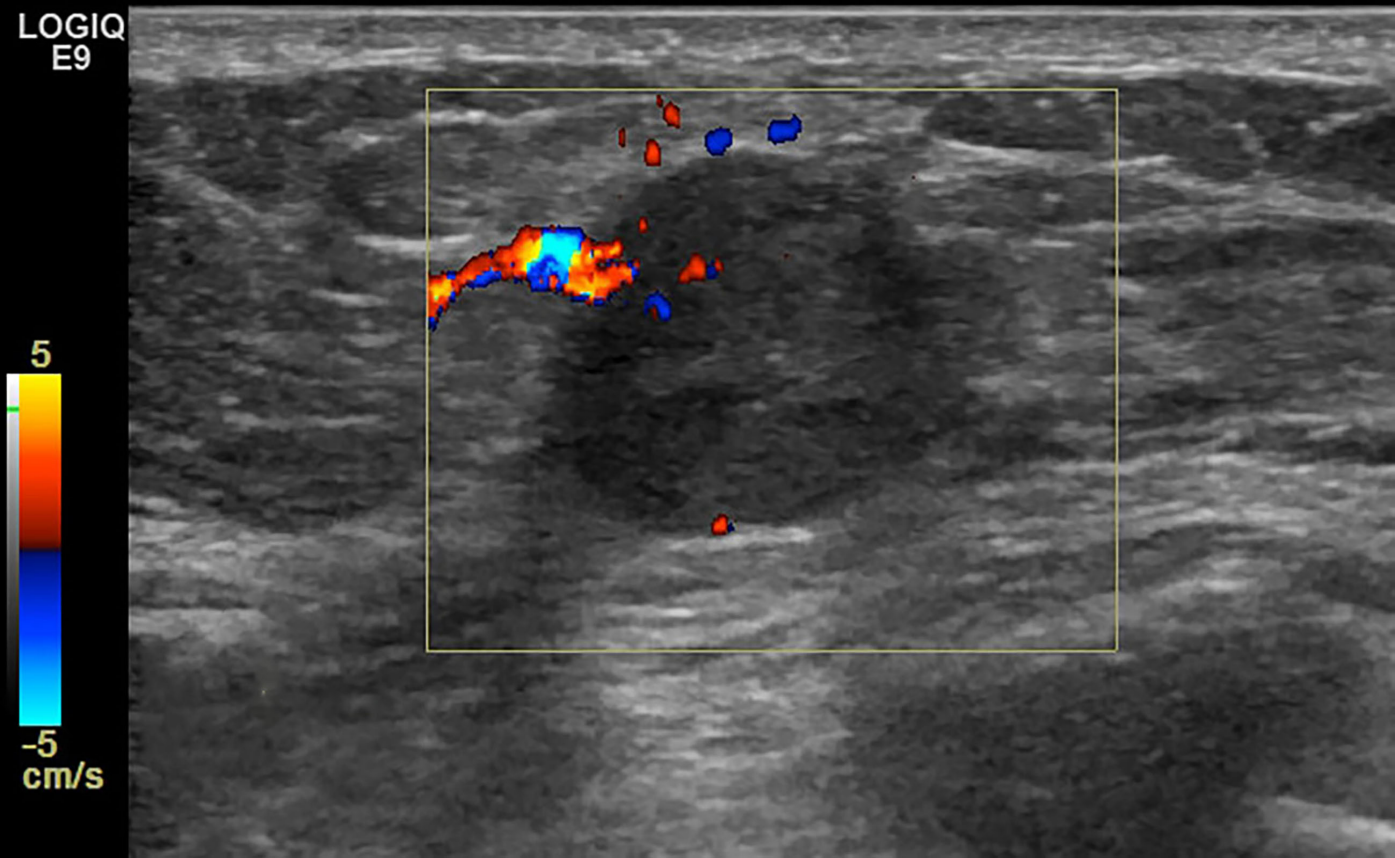
Case 3: A 49-year-old woman with cPMBC presented with a hypoechoic mass (1.7 × 1.4 × 1.3 cm) in the right breast. The lesion presented with linear blood flow signal by color Doppler mode.

The 133 lesions consisted of 11 MUMPCs, 65 cPMBCs, and 57 MMBCs. Compared with cPMBC, non-circumscribed margin (**Figure 1**), irregular shape (**Figure 2**), invasive growth (**Figure 3**), and lymph node metastasis occurred more frequently in MUMPC (100% *vs*. 58.5%, 100% *vs*. 67.7%, 100% *vs*. 58.5%, 72.7% *vs*. 24.6%, respectively, *p* < 0.05, **Table 1**). Irregular shape, microcalcification (**Figure 4**), invasive growth, and lymph node metastasis occurred more frequently in MMBC (89.5% *vs*. 67.7%, 50.9% *vs*. 26.2%, 78.9% *vs*. 58.5%, and 57.9% *vs*. 24.6%, respectively, *p* < 0.05, **Table 2**). Posterior acoustic enhancement (**Figure 5**) and missed diagnosis (33.3% *vs*. 58.5% and 1.8% *vs*. 18.5%, respectively, *p* < 0.05) were less frequent in MMBC. In patients aged >45 years, the difference in tumor size among the three groups was significant (*p* = 0.045). Paired comparisons showed that the difference in tumor size between cPMBC and MMBC was statistically significant (*p* = 0.014, **Figure 6**).

**Table 1:** Clinical characteristics of MUMPC, cPMBC and MMBC.

Parameters	MUMPC (*n* = 11)	cPMBC (*n* = 65)	MMBC (*n* = 57)	*p* value
Average age	53.73 ± 16.30	51.80 ± 15.56	52.61 ± 12.86	0.900^a^
Average size (cm)	2.65 ± 1.20^A,B^	2.88 ± 1.41^A^	2.21 ± 1.04^B^	0.014^a^
Soft	0	10 (15.4)	3 (5.3)	0.121^b^
Initial symptom				0.376^b^
Nipple discharge	1 (9.1)	2 (3.1)	2 (3.5)	
Palpable mass	10 (90.0)	60 (92.3)	55 (96.5)	
Asymptomatic	0	3 (4.6)	0	
Lymph node status				0.003^b^
Negative	3 (27.3)^B^	49 (75.4)^A^	33 (57.9)^A,B^	
Positive	8 (72.7)^B^	16 (24.6)^A^	24 (42.1)^A,B^	

Except where indicated, data are numbers of patients, with percentages in parentheses. Paired comparisons were conducted according to Bonferroni correction or Student–Newman–Keuls q test. If the superscript uppercase letters (^A,B^) of any two groups do not consist of the same letter, the difference between the two groups is considered to be significant.

a Data were analyzed by one-way ANVOA.

b Data were analyzed by Fisher’s exact test.

**Table 2:** Sonographic features of the MUMPC, cPMBC, and MMBC.

Parameters	*κ* coefficient	MUMPC (*n* = 11)	cPMBC (*n* = 65)	MMBC (*n* = 57)	*p*-value
Non-circumscribed	0.83	11 (100)^A^	38 (58.5)^B^	44 (77.2)^A,B^	0.004^a^
Irregular shape	0.85	11 (100)^A,B^	44 (67.7)^B^	51 (89.5)^A^	0.002^a^
Hypoechoic/isoechoic	0.87	9 (81.8)	54 (83.1)	47 (82.5)	>0.999
Microcalcification	0.95	6 (54.5)^A,B^	17 (26.2)^B^	29 (50.9)^A^	0.012^a^
Posterior acoustic enhancement	0.85	3 (27.3)^A,B^	38 (58.5)^B^	19 (33.3)^A^	0.010^a^
Invasive growth^b^	0.89	11 (100)^A^	38 (58.5)^B^	45 (78.9)^A^	0.003^a^
Abundant blood flow^c^	0.97	3 (27.3)	10 (15.4)	14 (24.6)	0.105^d^
Missed diagnosis		1 (9.1)^A,B^	12 (18.5)^B^	1 (1.8)^A^	0.006^a^

Except where indicated, data are numbers of patients, with percentages in parentheses. Paired comparisons were conducted according to Bonferroni correction or Student–Newman–Keuls q test. If the superscript uppercase letters (^A,B^) of any two groups do not consist of the same letter, the difference between the two groups is considered to be significant.

a Data were analyzed by Fisher’s exact test.

b Subcutaneous or/and retromammary fat layer were infiltrated.

c Adler blood flow grading II or III.

d Data were analyzed by the Kruskal–Wallis test.

## Discussion

MUMPC is a rare histological form of infiltrating breast carcinoma with estrogen receptor (ER) positivity, which accounts for <1% of breast cancers (8). MUMPC consists of micropapillary clusters of tumor cells with scalloped edges floating in stromal mucin (10). Compared with PMBC, MUMPC has a higher rate of lymph node metastasis and an outcome intermediate between that of mucinous carcinoma and micropapillary carcinoma (MPC) (11). MUMPC was described as a micropapillary variant of PMBC by Ng in 2002 (12). After that, a few researchers revealed that the incidence of MUMPC in PMBC was 12%–35% (8, 12, 13). The wide range may be ascribed to sampling bias and different diagnostic criteria. In the present study, the incidence was 14%, which accorded with previous studies.

In the present study, a non-circumscribed margin was significantly more likely to be found in MUMPC than in cPMBC (100% *vs*. 58.5%). In another study conducted by Zhang et al., the difference in margin was not significant (circumscribed *vs*. non-circumscribed) (14). The cause may be that interobserver agreement for tumor margin is low compared with shape, orientation, and echo pattern (15, 16). Therefore, misinterpretation of margin status may lead to a significant difference between the two groups.

The irregular shape is often considered to be an imaging feature associated with clinical prognosis. Lam et al. showed that irregular shape on sonographic imaging might be an indicator of unfavorable prognosis (17). Shet and Chinoy showed that the micropapillary subtype of mucinous cancer affected patient survival *via* its propensity for lymph node metastasis, depending on the amount of mucin within the tumor, irregularity of the tumor border, and tumor stage (13). In our study, irregular shape (lobulated or polygonal) could be identified in 100% of MUMPC and 67.7% of cPMBC, and the difference between the two groups was significant. In the study of Kaoku et al., the irregular shape was found in 90.9% (10/11) of PMBCs (18), which was higher than in our study. This may have been because the sample size in their study was smaller, and the sampling bias may have led to the lower irregular shape ratio in our study.

Calcification in breast tumors is commonly considered to be an important basis of diagnostic imaging. In general, coarse calcification is indicative of benign tumors, while microcalcification is associated with malignant tumors. The study of Li et al. showed that malignant calcifications were associated with clinical or pathologic features of poor prognosis (19). In our study, the occurrence of microcalcification in cPMBC was lower than that in MUMPC and MMBC (26.2% *vs*. 54.5% and 50.9%, respectively). The difference in microcalcification between cPMBC and MMBC was significant. This result is consistent with the report of Liu et al. that calcification was rare in PMBC (20). This phenomenon may be related to the proportion of cancer cells and stroma within the tumor. PMBC consists of abundant mucin, so calcification occurs less frequently in PMBC.

Internal and posterior echo patterns are both crucial imaging features. We found that most of the tumors in the three groups were hypoechoic or isoechoic. Kaoku et al. reported that the percentage was 100% (11/11), and they also reported that the more cancer cells and stroma were contained within the tumor, the higher the level of internal echo (18), which explains why the proportion of lesions with posterior acoustic enhancement was higher in cPMBC than in MMBC(58.5% *vs*. 33.3%). The posterior acoustic pattern is certainly beneficial for distinguishing PMBC from MMBC, but the contrary is the case for MUMPC and cPMBC.

The color flow signal on sonography within the tumor is related to vascularity. In our study, 27.3% (3/11) of MUMPC presented with rich intralesional vascularization (color flow grade II or III), compared with 15.4% in PMBC and 14% in MMBC. The difference among the three groups was not significant. The blood supply of MBC was not rich. Abundant mucin occupied a large part of the gross tumor volume, which demonstrated the insufficiency of vascularization.

In addition to the sonographic features concerning malignant tumors, we also discussed and compared some clinical and demographic features such as age, tumor size, initial symptom, and lymph node status. In a previous study conducted by Kim et al., the mean age of patients with MUMPC was 53.9 years (9). Shet et al. reported that most patients with MUMPC were older than 41 years but younger than 60 years (13). In our study, the mean age of patients with MUMPC was 52.9 years, which is consistent with the study of Kim et al. (9).

**Figure 7:**
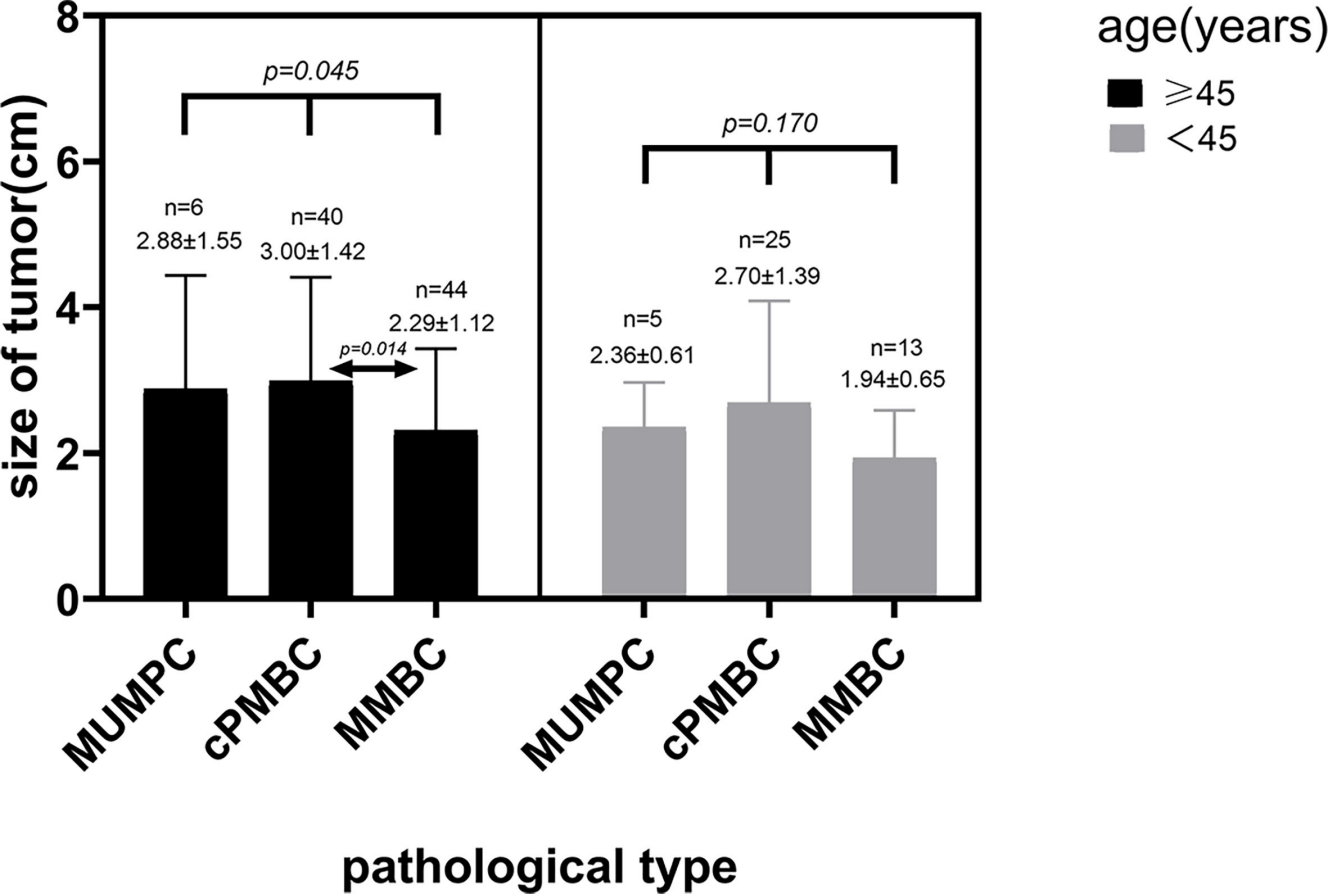
Distribution of tumor size in different pathological subtypes in different age groups.

In terms of tumor size, Lin et al. showed that the mean tumor size in MUMPC at diagnosis was 3.2 cm (range 0.8–9.0 cm) (21). In our study, the mean tumor size was 2.57 cm, which was in accordance with Lin et al. The difference in tumor size among the three pathological subtypes was significant (*p* = 0.014), and paired comparisons showed there was a significant difference between cPMBC and MMBC. We considered that age may be a confounding factor that may conceal the real correlation between tumor size and pathological type. In patients aged >45 years, the difference in tumor size among MUMPC, cPMBC, and MMBC was significant, and the mean tumor size in cPMBC was significantly larger than that in MMBC (**Figure 7**). The cause may be that the rate of missed diagnosis in cPMBC was larger than that in MMBC, and delayed diagnosis of cPMBC led to larger tumor size. In patients aged <45 years, the difference in tumor size between cPMBC and MMBC was not significant. This may be because patients aged <45 years seemed to be more health-conscious. They might choose to receive minimally invasive surgery to remove those benign-appearing lesions that were subsequently confirmed to be cPMBC pathologically. For patients aged >45 years, especially those who were elderly and poor, minimally invasive surgery was not widely accepted. Therefore, benign-appearing cPMBCs might be misdiagnosed and cancer diagnosis is delayed.

Lymph node status is a key factor affecting the prognosis of breast cancer. Previous studies showed that the metastatic lymph node ratio of MUMPC was 20%–42.9% (8–10, 12, 13). Liu et al. suggested that in MUMPC, lymphatic involvement was more frequent than in PMBC (22). Nevertheless, the lymphatic metastasis ratio of MUMPC was similar to that of MMBC (9). Our results were in line with previous studies, in which lymphatic metastasis was more likely in MUMPC than cPMBC (72.7% *vs*. 24.6%). The difference between MUMPC and MMBC was not significant (72.7% *vs*. 57.9%). The micropapillary pattern could harm the prognosis of PMBC.

We also noted that most MBC patients came to the hospital with a palpable breast mass as the initial symptom. There were only five cases of MBC with nipple discharge. Likewise, in a previous study conducted by Lee et al., 87% of MBC cases had a palpable mass (16). The abovementioned phenomena indicated that there were no specific signs and symptoms related to MBC, and age-appropriate follow-up ultrasound examination is still an effective method to detect MBC.

Our study had several limitations. First, all ultrasound images were two-dimensional, which might be deficient in carrying out an adequate and valid assessment. Second, this was a single-center study, and the sample size was not very large. MBC is a rare pathological subtype, and the MUMPC is even more infrequent than MBC. There were only 11 MUMPCs in this study. A future study should include a large sample size, especially of MUMPC.

## Conclusion

MUMPC commonly manifests as an irregular and parallel lesion on ultrasonography with a non-circumscribed or microlobulated margin, little microcalcification, and vascularity. It mainly manifests as a hypoechoic mass, with some complex lesions with cystic and solid components. Local infiltrative growth and regional lymphatic involvement are often seen in MUMPC with a lower misdiagnosis rate. Ultrasound is helpful to distinguish MUMPC from cPMBC.

## Data Availability Statement

The original contributions presented in the study are included in the article/**Supplementary Material**. Further inquiries can be directed to the corresponding author.

## Author Contributions

Conceptualization: WZ. Data curation: D-MC. Methodology: Y-ZL. Resources: WZ and L-MG. Writing—original draft: WZ. Writing—review and editing: D-MC. All authors contributed to the article and approved the submitted version.

## Conflict of Interest

The authors declare that the research was conducted in the absence of any commercial or financial relationships that could be construed as a potential conflict of interest.

## Publisher’s Note

All claims expressed in this article are solely those of the authors and do not necessarily represent those of their affiliated organizations, or those of the publisher, the editors and the reviewers. Any product that may be evaluated in this article, or claim that may be made by its manufacturer, is not guaranteed or endorsed by the publisher.

## References

[1] Di Saverio S Gutierrez J Avisar E. A Retrospective Review With Long Term Follow Up of 11,400 Cases of Pure Mucinous Breast Carcinoma. Breast Cancer Res Treat (2008) 111:541–7. doi: 10.1007/s10549-007-9809-z 18026874

[2] Chaudhry AR El Khoury M Gotra A Eslami Z Omeroglu A Omeroglu-Altinel G. Imaging Features of Pure and Mixed Forms of Mucinous Breast Carcinoma With Histopathological Correlation. Br J Radiol (2019) 92:20180810. doi: 10.1259/bjr.20180810 30632779 PMC12187173

[3] Rosen PP Lesser ML Kinne DW. Breast Carcinoma at the Extremes of Age: A Comparison of Patients Younger Than 35 Years and Older Than 75 Years. J Surg Oncol (1985) 28:90–6. doi: 10.1002/jso.2930280204 2982064

[4] Yang WT Zhu XZ. The Introduction of 2012 WHO Classification of Tumours of the Breast. Zhonghua Bing Li Xue Za Zhi (2013) 42:78–80. doi: 10.3760/cma.j.issn.0529-5807.2013.02.002 23710911

[5] Kashiwagi S Onoda N Asano Y Noda S Kawajiri H Takashima T. Clinical Significance of the Sub-Classification of 71 Cases Mucinous Breast Carcinoma. Springerplus (2013) 2:481. doi: 10.1186/2193-1801-2-481 24156087 PMC3797911

[6] Barkley CR Ligibel JA Wong JS Lipsitz S Smith BL Golshan M. Mucinous Breast Carcinoma: A Large Contemporary Series. Am J Surg (2008) 196:549–51. doi: 10.1016/j.amjsurg.2008.06.013 18809061

[7] Dumitru A Procop A Iliesiu A Tampa M Mitrache L Costache M. Mucinous Breast Cancer: A Review Study of 5 Year Experience From a Hospital-Based Series of Cases. Maed (Bucur) (2015) 10:14–8. PMC4496759 26225144

[8] Barbashina V Corben AD Akram M Vallejo C Tan LK. Mucinous Micropapillary Carcinoma of the Breast: An Aggressive Counterpart to Conventional Pure Mucinous Tumors. Hum Pathol (2013) 44:1577–85. doi: 10.1016/j.humpath.2013.01.003 23517923

[9] Kim HJ Park K Kim JY Kang G Gwak G Park I. Prognostic Significance of a Micropapillary Pattern in Pure Mucinous Carcinoma of the Breast: Comparative Analysis With Micropapillary Carcinoma. J Pathol Transl Med (2017) 51:403–9. doi: 10.4132/jptm.2017.03.18 PMC5525037 28597867

[10] Ranade A Batra R Sandhu G Chitale RA Balderacchi J. Clinicopathological Evaluation of 100 Cases of Mucinous Carcinoma of Breast With Emphasis on Axillary Staging and Special Reference to a Micropapillary Pattern. J Clin Pathol (2010) 63:1043–7. doi: 10.1136/jcp.2010.082495 20962055

[11] Pareja F Selenica P Brown DN Sebastiao A da Silva EM Da Cruz Paula A. Micropapillary Variant of Mucinous Carcinoma of the Breast Shows Genetic Alterations Intermediate Between Those of Mucinous Carcinoma and Micropapillary Carcinoma. Histopathology (2019) 75:139–45. doi: 10.1111/his.13853 PMC6591080 30843622

[12] Ng WK. Fine-Needle Aspiration Cytology Findings of an Uncommon Micropapillary Variant of Pure Mucinous Carcinoma of the Breast: Review of Patients Over an 8-Year Period. Cancer (2002) 96:280–8. doi: 10.1002/cncr.10747 12378595

[13] Shet T Chinoy R. Presence of a Micropapillary Pattern in Mucinous Carcinomas of the Breast and its Impact on the Clinical Behavior. Breast J (2008) 14:412–20. doi: 10.1111/j.1524-4741.2008.00616.x 18673338

[14] Zhang H Qiu L Peng Y. The Sonographic Findings of Micropapillary Pattern in Pure Mucinous Carcinoma of the Breast. World J Surg Oncol (2018) 16:151. doi: 10.1186/s12957-018-1449-8 30041628 PMC6058370

[15] Lazarus E Mainiero MB Schepps B Koelliker SL Livingston LS. BI-RADS Lexicon for US and Mammography: Interobserver Variability and Positive Predictive Value. Radiology (2006) 239:385–91. doi: 10.1148/radiol.2392042127 16569780

[16] Lee HJ Kim EK Kim MJ Youk JH Lee JY Kang DR. Observer Variability of Breast Imaging Reporting and Data System (BI-RADS) for Breast Ultrasound. Eur J Radiol (2008) 65:293–8. doi: 10.1016/j.ejrad.2007.04.008 17531417

[17] Lam WW Chu WC Tse GM Ma TK. Sonographic Appearance of Mucinous Carcinoma of the Breast. AJR Am J Roentgenol (2004) 182:1069–74. doi: 10.2214/ajr.182.4.1821069 15039190

[18] Kaoku S Konishi E Fujimoto Y Tohno E Shiina T Kondo K. Sonographic and Pathologic Image Analysis of Pure Mucinous Carcinoma of the Breast. Ultrasound Med Biol (2013) 39:1158–67. doi: 10.1016/j.ultrasmedbio.2013.02.014 23683410

[19] Li JN Xu J Wang J Qing C Zhao YM Liu PF. Correlation Between Mammograghic Findings and Clinical/Pathologic Features in Women With Small Invasive Breast Carcinomas. Asian Pac J Cancer Prev (2014) 15:10643–6. doi: 10.7314/apjcp.2014.15.24.10643 25605153

[20] Liu H Tan H Cheng Y Zhang X Gu Y Peng W. Imaging Findings in Mucinous Breast Carcinoma and Correlating Factors. Eur J Radiol (2011) 80:706–12. doi: 10.1016/j.ejrad.2010.06.008 20615642

[21] Lin HY Gao LX Jin ML Ding HY. Clinicopathologic Features of Micropapillary Variant of Pure Mucinous Carcinoma of Breast. Zhonghua Bing Li Xue Za Zhi (2012) 41:613–7. doi: 10.3760/cma.j.issn.0529-5807.2012.09.009 23157830

[22] Liu F Yang M Li Z Guo X Lin Y Lang R. Invasive Micropapillary Mucinous Carcinoma of the Breast Is Associated With Poor Prognosis. Breast Cancer Res Treat (2015) 151:443–51. doi: 10.1007/s10549-015-3413-4 25953688

