# Erratum: Sonographic Features of Pure Mucinous Breast Carcinoma With Micropapillary Pattern

**DOI:** 10.3389/fonc.2021.822462

**Published:** 2021-12-13

**Authors:** Frontiers Production Office

**Affiliations:** Frontiers Media SA, Lausanne, Switzerland

**Keywords:** breast, pure mucinous carcinoma, micropapillary, ultrasonography, mixed mucinous carcinoma

Due to a production error, the title was incorrectly written as “Sonographic Features of Pure Mucinous Brelast Carcinoma With Micropapillary Pattern”. The correct title is “Sonographic Features of Pure Mucinous Breast Carcinoma With Micropapillary Pattern”.

The publisher apologizes for this mistake. The original version of this article has been updated.

